# Supplementary material for: Research data management in agricultural sciences in Germany: We are not yet where we want to be
Source: PLoS One. 2022 Sep 30;17(9):e0274677. doi: 10.1371/journal.pone.0274677 (PMC9524626; doi:10.1371/journal.pone.0274677)
Supplement: Attachment — Submitted filename: Response to Reviewers.pdf [file pone.0274677.s001.pdf]

Datenverarbeitung und IT
Data processing department

Julius Kühn-Institut
Bundesforschungsinstitut für Kulturpflanzen
Federal Research Centre for Cultivated Plants

JKI, Stahnsdorfer Damm 8, 14532 Kleinmachnow, Germany

www.julius-kuehn.de

To
Prof. Dr. Cataldo Pulvento
Academic Editor
PLOS ONE

Dr. Matthias Senft
Research Data Management

Fon: +49 3946 47 2442
Fax: +49 33203 48-425

E-Mail: matthias.senft@julius-kuehn.de

Potsdam, 28th July 2022

Response to Reviewers (PONE-D-21-39572)

Manuscript Number: PONE-D-21-39572
Initial Title: Research Data Management in Agricultural Sciences: We are not yet where we want to be.
Authors: Matthias Senft, Ulrike Stahl and Nikolai Svoboda
Status: Minor Revision

Reviewer's Comments and Suggestions

- our responses to each point are colored in blue -

Reviewer #1

This manuscript describes and interprets the results of a survey, conducted in 2020, regarding publication, description, and reuse of data in the agricultural sciences. I think that the paper describes important challenges in data management. It presents and interprets the results of the survey straightforwardly. There is not a lot of complexity in the paper to comment on.

The writing is somewhat challenging to parse in some places. Editing for clarity and concision would help. I list a few examples here:

Lines 102-103: Something is missing or extraneous in this sentence: "In order to be able to build these infrastructures along the specific requirements of the users must first be evaluated." Perhaps it should be "In order to be able to build these infrastructures, the specific requirements of the users must first be evaluated."

→ L109-110: We rewrote the sentence: „In order to be able to build such an infrastructure, the specific requirements of the users must first be evaluated.“

Lines 103-108: This is a long sentence that terminates in a list. The list elements are non-parallel. It would be helpful to break this into two sentences. Also, I am not sure that "data publishing is good scientific practice" is an independent reason. I mean: following FAIR principles is good to do because it is good scientific practice ... is tautological.

→ L110-116: As suggested we broke the sentence into two and changed the listing: „Especially in the domain of agricultural sciences, there is no common understanding among typical users what RDM following the FAIR principles means [19]. Often, the mutual benefits of FAIR data provision remains unrecognized by both data creators and (re-)users [24;25]: more and secured storage, time and cost savings, facilitating validation, reproducibility and reuse consequently avoiding duplicative studies and accelerating research, increasing citability and reputation. Additionally, it is increasingly requested by funding agencies and becomes a prerequisite of data papers or scientific articles.“

Line 127: "In order to develop an RDM along the needs," ... needs of what?

→ L 136: We added “of involved user groups“ to that sentence.

Lines 471-473: This sentence would be more easily parsed if the verb were closer to the subject:
"In a first step, awareness must be raised regarding the need for good RDM and ultimately the availability and use of standardized data sets."

→ L505-507: We changed the sentence as suggested.

***Reviewer #2**:*

Summary: The reviewed study entitled *Research Data Management in Agricultural Sciences: We are not yet where we want to be.* assesses the broad state of data management practices in the crop science community with a survey-based approach. The authors highlight obstacles and challenges limiting data circulation and propose ways forward for building better infrastructure and services in the agricultural sciences field.

Review: The paper is solid, clearly written and focused on a precise question on a key matter not often addressed within the crop science community. The survey and its analysis enabled the authors to draw a clear picture of the concerned data type, and data sharing and discovery practices. The authors conclude that education on better data management practices is essential in raising awareness and advocating for better technical innovations (standardization, distribution). The results support the claims of the authors and the discussion is careful.

General comments:

At this step, I don't have precise questions that should be addressed as a prerequisite to the publication of this work. However, I propose a few comments that might add a bit of nuance to the message in this paper.

The first comment is about the validity of the analysis outside Germany. I understand that all the answers to the online survey came from various academic or extension or industry structures in Germany. Maybe the authors could add the country name in the title, and add a sentence that could help readers to generalize the results at least to Europe?

→ Lines 4-5: As suggested, we added „in Germany“ to the title.

→ Lines 543-547: We added the following sentences to the end of the conclusion: „Although this survey was conducted on a national level in Germany, the results of this study can help to address overarching challenges in RDM and professionalize it on an international level as well. RDM is not a national concern, because only together the common

challenges in RDM can be solved and will advance global research in agricultural sciences making it at the same time more transparent and efficient."

My second comment is about the discussion, more precisely about the way forward to improve research data management. I completely agree with the need to improve the value of experimental data by promoting its reuse. The authors mention education and technical solutions as key levers. However, this section could be enriched with a few insights from literature, to illustrate that acting at a small scale, without aiming for a perfect set of RDM practices might initiate change.

- For example, in *Good enough practices in scientific computing* (https://doi.org/10.1371/journal.pcbi.1005510), Wilson et al. explain that a minimum set of tools and techniques ("good enough" practices) is a first step before trying to set up standards and platforms.
- * in *Our path to better science in less time using open data science tools* (https://doi.org/10.1038/s41559-017-0160) Lowndes et al. how open data science tools help to drive a better science, starting at the lab scale.

→ Lines 530-536: We added a few sentences including the two suggested references to the manuscript: „Once the various tools are available, findable, usable and finally open to the scientific community, the first step would be to familiarize scientists with the tools and motivate them to use tailored tools. Past experience has shown that this is better done in small steps [61,62]. As perfectly said by Lowndes et al., 'engagement may best be approached as an evolution rather than as a revolution that may never come' [61]. Therefore, the aim should be to establish a minimum set of tools and techniques first rather than directly aiming to establish perfect RDM practices at once [61,62]."

Suggestions:

> l51. However, state-of-the-art research data management and a clever combination of various useful data offer the potential to move from a GHG source to a carbon sink.
In the introduction section, I suggest toning down this bold assertion, I think that such broad transitions in agricultural systems are not only a problem of research data management, and necessitate multiple points of view on knowledge production. For example, in *A tool for reflecting on research stances to support sustainability transitions* (https://doi.org/10.1038/s41893-019-0440-x) Hazard et al. illustrate how the positivist, reductionist and instrumental stance has proved their ability to solve well-defined problems but that more complex issues, such as sustainability transitions require a scientific pluralism.

→ Lines 57-59: As suggested, we toned that assertion down a bit: „However, state of the art research data management and clever combination of various useful data can, among other things, help to move from a GHG source to a carbon sink."

Journal Requirements

1. Please ensure that your manuscript meets PLOS ONE's style requirements, including those for file naming. The PLOS ONE style templates can be found at https://journals.plos.org/plosone/s/file?id=wjVg/PLOSOne_formatting_sample_main_body.pdf and https://journals.plos.org/plosone/s/file?id=ba62/PLOSOne_formatting_sample_title_authors_affiliations.pdf

→ We adapted respective parts of the manuscript to meet PLOS ONE's style requirements.

2. Please review your reference list to ensure that it is complete and correct. If you have cited papers that have been retracted, please include the rationale for doing so in the manuscript text, or remove these references and replace them with relevant current references. Any changes to the reference list should be mentioned in the rebuttal letter that accompanies your revised manuscript. If you need to cite a retracted article, indicate the article's retracted status in the References list and also include a citation and full reference for the retraction notice.

→ We checked the reference list and updated URL availability and made smaller changes to meet the reference guidelines.
→ We corrected reference [25] as we previously referenced to the F1000Reseach review instead to the actual F1000Reseach paper.
→ We updated reference [40] as it was no longer available at the previously provided URL.
→ We updated reference [53] as the referenced infrastructure was renamed („DaCuRa" -> „DataCube") and also changed its URL, we updated that also in the text (L477).
→ We added two references suggested by Reviewer 2:

> [61] Lowndes JSS, Best BD, Scarborough C, Afflerbach JC, Frazier MR, O'Hara CC, Jiang N, Halpern BS. Our path to better science in less time using open data science tools. Nat Ecol Evol. 2017; 1, 0160. doi: 10.1038/s41559-017-0160.
>
> [62] Wilson G, Bryan J, Cranston K, Kitzes J, Nederbragt L, Teal TK. Good enough practices in scientific computing. PLoS computational biology. 2017; 13(6), e1005510. doi: 10.1371/journal.pcbi.1005510.

→ We added one more references (L539-540) as it was published only recently:

> [63] Deutsche Forschungsgemeinschaft | AG Publikationswesen. Wissenschaftliches Publizieren als Grundlage und Gestaltungsfeld der Wissenschaftsbewertung. 2022. doi:10.5281/zenodo.6538163.

While revising your submission, please upload your figure files to the Preflight Analysis and Conversion Engine (PACE) digital diagnostic tool, https://pacev2.apexcovantage.com/. PACE helps ensure that figures meet PLOS requirements. To use PACE, you must first register as a user. Registration is free. Then, login and navigate to the UPLOAD tab, where you will find detailed instructions on how to use the tool. If you encounter any issues or have any questions when using PACE, please email PLOS at figures@plos.org. Please note that Supporting Information files do not need this step.

→ We uploaded all figures to PACE and converted them from eps to tif format. There seemed to be everything fine with them. We uploaded the PACE corrected version together with the revision.

Explanation for Additional Changes

Changed contact information of the corresponding author

→ The corresponding author changed employers in April 2022. We added the current address on the title page to the corresponding author.
